# Synergistic anti-tumor activity of the mTOR inhibitor everolimus and gemcitabine for relapsed/refractory peripheral T cell lymphoma

**DOI:** 10.3389/fimmu.2025.1683550

**Published:** 2025-11-28

**Authors:** Ling Huang, Hanguo Guo, Yan Teng, Diwen Pang, Fen Zhang, Yichen Sun, Choon Kiat Ong, Jing Tan, Wenyu Li

**Affiliations:** 1 Lymphoma Division, Guangdong Provincial People’s Hospital (Guangdong Academy of Medical Sciences), Southern Medical University, Guangzhou, China; 2 School of Medicine, South China University of Technology, Guangzhou, China; 3 Department of Pathology, Guangdong Provincial People’s Hospital (Guangdong Academy of Medical Sciences), Southern Medical University, Guangzhou, China; 4 State Key Laboratory of Oncology in South China, Collaborative Innovation Center of Cancer Medicine, Sun Yat-sen University Cancer Center, Guangzhou, China; 5 Cancer and Stem Cell Biology Program, Duke-NUS Medical School, Singapore, Singapore; 6 Lymphoma Genomic Translational Research Laboratory, Cellular and Molecular Research, National Cancer Centre Singapore, Singapore, Singapore

**Keywords:** relapsed/refractory, peripheral T-cell lymphoma (PTCL), everolimus, gemcitabine, synergistic, anti-tumor activity

## Abstract

**Background:**

Relapsed/refractory (R/R) peripheral T-cell lymphoma (PTCL) patients face poor prognosis and limited therapies. The mTOR inhibitor everolimus and gemcitabine show modest efficacy as single agents in R/R PTCL, warranting investigation of combination regimens.

**Methods:**

This study conducted a single-center retrospective analysis of 24 patients diagnosed with peripheral T-cell lymphoma, aged 18 to 70 years, who experienced relapse or refractory disease following at least one first-line chemotherapy regimen, and received treatment at Guangdong Provincial People’s Hospital between December 2017 and March 2021. CellTiter Glo and AnnexinV FITC/PI assays evaluated cell viability and apoptosis *in vitro*.

**Results:**

The combination showed significant efficacy: objective response rate (ORR) 70.8%, complete response (CR) rate 45.8%, median time to response 1.7 months. With median follow-up of 22.3 months (95% CI 5.7–39.0), median progression-free survival (PFS) was 9.9 months (95% CI 0–20.81), and median duration of response (DOR) 16.8 months. Patients with CR/partial response (PR) had longer PFS (18.7 vs. 1.2 months, *P* = 0.0007) and overall survival (OS) (30.9 vs. 5.8 months, *P* = 0.021) than those with stable/progressive disease. RNA-seq results showed that the combined therapeutic approach synergistically reduces cell viability and promotes apoptosis through the inhibition of the MYC signaling pathway.

**Conclusion:**

Everolimus-gemcitabine combination exhibits synergistic antitumor activity, offering a potential therapeutic strategy for R/R PTCL.

## Introduction

Peripheral T-cell lymphoma (PTCL) comprises a rare and heterogeneous group of mature T-cell neoplasms, accounting for ~15% of non-Hodgkin lymphomas (NHL) (1). The 2016 World Health Organization classification defines 29 PTCL subtypes, including PTCL-not otherwise specified (PTCL-NOS), angioimmunoblastic T-cell lymphoma (AITL), anaplastic lymphoma kinase (ALK) positive anaplastic large cell lymphoma (ALK+ALCL), ALK negative ALCL (ALK-ALCL) and others (2). While first-line therapy predominantly involves CHOP (cyclophosphamide, doxorubicin, vincristine, and prednisone) regimens, the prognosis for the majority of peripheral T-cell lymphoma (PTCL) subtypes remains unfavorable, except for ALK-positive anaplastic large cell lymphoma. Specifically, excluding ALK-positive anaplastic large cell lymphoma, the overall prognosis for patients with PTCL is generally poor, as approximately only 30% of patients achieve cure following initial treatment (3). For relapsed/refractory (R/R) PTCL patients, the median progression-free survival (PFS) and overall survival (OS) are merely 3.1 and 5.5 months, respectively (3). Most recently approved therapeutic agents for relapsed or refractory peripheral T-cell lymphoma (R/R PTCL) demonstrate overall response rates (ORR). Brentuximab vedotin (BV) is an antibody-drug conjugate specifically directed against the CD30 antigen. It leverages the targeting specificity of the monoclonal antibody to facilitate localized delivery and release of the cytotoxic agent within tumor tissues, thereby integrating the therapeutic effects of both the antibody and the anti-cancer drug. Clinical investigations have demonstrated that brentuximab vedotin attains an objective response rate of 41% in patients with CD30-positive non-invasive large cell lymphoma, excluding anaplastic large cell lymphoma (non-ALCL) subtypes (4–7). Thus, well-tolerated combination therapies targeting critical pathways are urgently needed.

Aberrant activation of the PI3K/AKT/mammalian target of rapamycin (mTOR) signaling pathway contributes to tumorigenesis, metastasis, chemoresistance, and poor outcomes in PTCL patients (8). Targeting this pathway has emerged as a promising therapeutic strategy (8, 9). Duvelisib is an innovative inhibitor targeting leukocyte signaling pathways, functioning by suppressing the activation of these pathways through the inhibition of multiple phosphoinositide 3-kinase (PI3K) isoforms. This mechanism effectively impedes the aberrant proliferation and dissemination of leukemia and lymphoma cells. Clinical investigations have revealed that duvelisib exhibits promising therapeutic efficacy in patients with relapsed or refractory peripheral T-cell lymphoma, achieving an overall response rate of 50%, alongside a favorable safety profile (10). Mechanistically, mTOR inhibition not only restricts tumor growth but also enhances T cell activation to augment tumor specific immunity, underscoring the therapeutic potential of mTOR inhibitor in T cell lymphomas (9). The mTOR inhibitor everolimus has shown an ORR of 44% and a complete response (CR) of 6.3%, with a median PFS of 4.1 months in R/R PTCL patients (11). However, single-agent everolimus fails to overcome therapeutic limitations in PTCL, necessitating combination approaches.

Gemcitabine-based salvage therapy has been established as an effective approach for R/R NHL. In R/R PTCL, single-agent gemcitabine achieves an ORR of 51% and a CR rate of 23% (12). Despite these results, the suboptimal efficacy highlights the need for innovative combination strategies. Preclinical studies have demonstrated synergistic antitumor activity of everolimus and gemcitabine in T cell lymphoma cell lines (13). Herein, we evaluated the clinical efficacy of this combination therapy in patients with relapsed/refractory peripheral T-cell lymphoma and validated its mechanism of action *in vitro*, thereby providing data to inform the optimization of clinical treatment regimens.

## Methods

### Patient cohort and treatment protocol

This retrospective study included 24 patients treated at Guangdong Provincial People’s Hospital between December 2017 and March 2021, who met the following criteria: aged 18 to 70 years, with histologically confirmed peripheral T-cell lymphoma, experiencing relapse at least one year after initial therapy (defined as disease recurrence more than one year from the start of treatment) or refractory disease (recurrence within one year of treatment initiation or disease progression during therapy). All patients consented to anti-tumor treatment, and those with severe infections, interstitial pneumonia, who declined chemotherapy, or were unable to take oral medications were excluded. Patient staging followed the Ann Arbor classification (14), with bulky disease defined as tumor mass ≥7.5 cm (15). Treatment response was evaluated via enhanced computed tomography (CT) or ^18^F-fluorodeoxyglucose positron emission tomography/CT (FDG-PET/CT) imaging after 2, 4 and 6 cycles according to the Lugano 2014 criteria (16). Clinical data were extracted from electronic medical records, and written informed consent was obtained from all patients.

The combination regimen consisted of gemcitabine (1,000 mg/m² on days 1 and 8) and oral everolimus (10 mg daily), administered every 21 days for up to 6 cycles. Patients achieving complete response (CR) after 4–6 cycles were considered for hematopoietic stem cell transplantation (HSCT), including autologous (ASCT) or allogeneic (allo-SCT) transplantation. Non-transplant-eligible patients continued everolimus monotherapy for 1 year. Supportive care included granulocyte-colony stimulating factor (G-CSF), thrombopoietin, erythropoietin, and blood/platelet transfusions as needed.

### Reagents and cell lines

Everolimus (RAD001), gemcitabine and BZE235 were purchased from Selleck Chemicals (Houston, USA). Antibodies against AKT (CST, #2920), p70S6K (CST, #2708), S6(CST, #2217), phospho-Ser473-AKT (CST, #4060), phospho-Thr308-AKT (CST, #2965), phospho-Thr421/Ser424-p70S6K (CST, #9204), phospho-Ser240/244-S6 (CST, #5364), poly (ADP-ribose) polymerase (PARP) (CST, #9542) and cleaved PARP (CST, #5625) were purchased from Cell Signalling Technologies (Danvers, MA, USA).

Nine human PTCL cell lines (SU-DHL-1, L-82, KI-JK, DEL, SR-786, KARPAS-299 and SUP-M2) were maintained in RPMI 1640 medium supplemented with 20% foetal bovine serum (FBS). FE-PD and SMZ-1 were kindly provided by Dr. Suzanne Turner (University of Cambridge) and Dr. Samuel Ng (Dana-Farber Cancer Institute), respectively; others were from DSMZ (Braunschweig, Germany).

### Immunohistochemistry

Formalin-fixed, paraffin-embedded, 4-μm tissue sections were used for immunohistochemistry of phosphorylated AKT(p-AKT) and phosphorylated p70S6 kinase(p-p70S6K). Primary antibodies specific to p-AKT (ser473) (CST, #4060,1:100) and p-p70S6K (Thr421/Ser424) (CST, #9204, 1:400) were used. Negative controls omitted primary antibodies, replacing them with phosphate-buffered saline (PBS). Slides were evaluated by a single pathologist blinded to clinical data.

### Construction of MYC overexpression cell line

To generate empty vector and MYC-overexpressing retroviruses for subsequent transduction of SU-DHL-1 cells, the constructed empty vector and MYC-overexpressing plasmids—generously provided by Prof. Jing Tan’s laboratory at Sun Yat-sen University Cancer Center—were transfected into Plat-A cells using Lipofectamine 2000 (Invitrogen) according to the manufacturer’s instructions. The culture medium was replaced 6 hours post-transfection, and retrovirus-containing supernatant was harvested at 48 hours after transfection. The collected viral supernatant was filtered through a 0.45 µm filter (Millex) and used to infect the target host cell line for 48 hours in the presence of 8 µg/µL polybrene (Sigma-Aldrich) to enhance infection efficiency. To establish stable cell lines expressing either the empty vector or MYC, GFP-positive cells were sorted based on green fluorescence (488 nm excitation) using a FACSAria III flow cytometer (BD Biosciences). Overexpression of MYC protein was further confirmed by Western blot analysis.

### *In vitro* functional assays

Cell Viability and Proliferation: Cells (5×10³/well) were seeded in 96-well plates and treated with everolimus, gemcitabine, or their combination for 96 hours (viability) or 4 days (proliferation). CellTiter Glo reagent (Promega) measured ATP levels to assess viability/proliferation, with half-maximal inhibitory concentration (IC_50_) calculated using GraphPad Prism (nonlinear regression, log(inhibitor) vs. response model).

Synergy Analysis: Combination index (CI) was determined via the Chou-Talalay method using CalcuSyn software, with CI < 1 indicating synergistic effects. The CI values were determined by the inhibition rate of the cells calculated with CalcuSyn software base on the Chou-Talalay Method for Anti-tumor Drug Combination (17).

Apoptosis assay: Apoptosis was assessed using the AnnexinV FITC/PI apoptosis kit (Invitrogen, Carlsbad, CA, USA) according to the manufacturer’s instructions, and the Spectra cell analyser (Sony Inc., Tokyo, Japan) as described previously (13).

Western Blotting: Cells were harvested, lysed with RIPA buffer and cleared of debris by centrifugation at 14000 rpm for 15 minutes. Protein concentration was measured by Quick StartTM Bradford Protein Assay (Bio-Rad), and 15 µg protein was loaded on SDS-PAGE and transferred to PVDF membrane. Membranes were blocked in 5% BSA in Tris-buffered saline (TBS, 10 mM Tris, 10 mM NaCl) and overnight incubation with primary antibodies. The secondary antibodies were HRP-conjugated anti-rabbit and anti-mouse (GE Healthcare, NA931 and NA934). Immunoblotting was developed with ECLTM Western Blotting Detection Reagents (GE Healthcare life science). Protein bands were visualized using ECL reagents and a Bio-Rad ChemiDoc MP system.

### RNA sequencing and transcriptomic analysis

RNAs were collected from SU-DHL-1 cells treated with DMSO, gemcitabine (4nM), everolimus (50nM) for 24 hours. Total RNA was extracted using Trizol Reagent (Invitrogen), following by RNeasy Mini Kit (Qiagen). Transcriptome sequencing libraries were prepared using TruSeq Stranded RNA HT kit (Illumina, 15032620) according to the manufacturer’s protocol. Briefly, Ribosomal RNA was removed by beads and RNA was fragmented into small pieces and reverse transcribed to single-strand cDNA. The cDNA was ligated to the adaptors and then amplified with the standard Illumina library preparation. Samples were sequenced on the Illumina NovaSeq6000 according to the manufacturer’s protocol. For data analysis, fastp software (version 0.12.5) was used to detect the quality of the original data and the adaptors were removed. All the clean reads were aligned to reference human genome (GRCh38, hg38) with STAR with GENCODE annotation (genocide. v37. annotation. gtf). RSEM was used to quantify gene expression levels. Essentially, edger (|log_2_FC| > 1 or < -1, *P* < 0.05) was used to identify differentially expressed genes (DEGs). DEGs were identified using edger (|log_2_FC| > 1, *p* < 0.05). Data are available at GEO (accession: GSE185604).

### Statistical analysis

Clinical outcomes—objective response rate (ORR), duration of response (DOR), progression-free survival (PFS), and overall survival (OS)—were defined per Lugano criteria. Survival curves were estimated via Kaplan-Meier analysis and compared using the log-rank test (GraphPad Prism 9.0). Adverse events were graded by CTCAE v5.0 (SPSS v13.0). Statistical significance was set at *p* < 0.05 (two-sided), with *p* values denoted as * (0.01–0.05), ** (0.001–0.01), *** (0.0001–0.001), or **** (< 0.0001).

## Results

### Clinical efficacy of everolimus-gemcitabine combination in R/R PTCL

**Figure 1:**
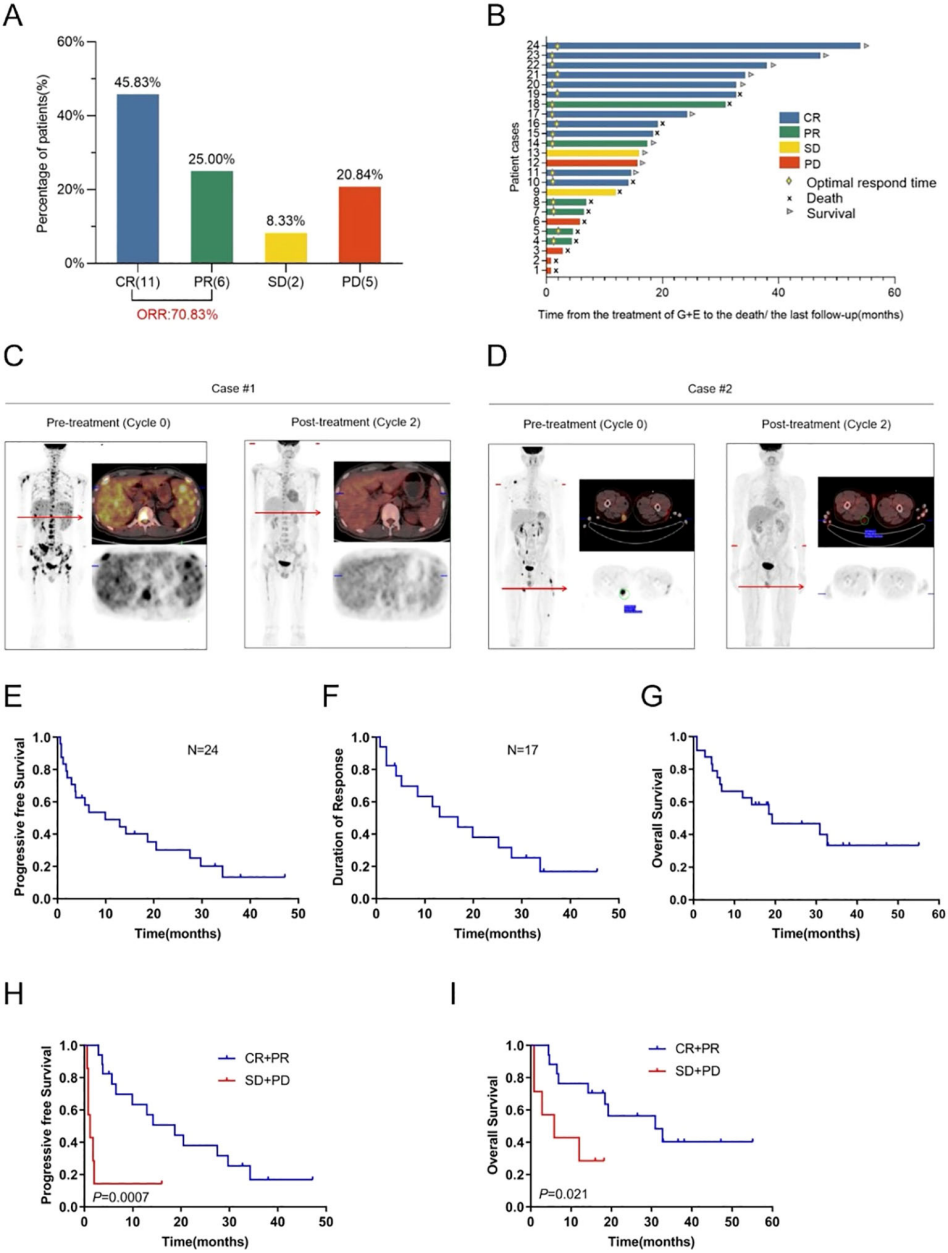
Clinical efficacy of combination regimen everolimus and gemcitabine in patients with R/R PTCL. **(A)** Best response in the 24 patients with R/R PTCL. The objective response rate (ORR) was 70.8%, with a complete response rate of 45.8%. **(B)** Bar diagram of the survival outcomes of the 24 patients. Eleven patients achieved CR: Patient#24 (ALK-ALCL), Patient#23 (AITL), Patient#22 (PTCL-NOS), Patient#21 (SPTCL), Patient#20 (ALK-ALCL), Patient#19 (PTCL-NOS), Patient#17 (PTCL-NOS), Patient#16 (SPTCL), Patient#15 (ALK-ALCL), Patient#11(ALK-ALCL), Patient#10 (PTCL-NOS). Patient#24, 23, 22, 19, 16, 15 and 10 underwent HSCT. **(C, D)** Indicators of the responses in two representative cases (two patients with PTCL-NOS). **(E)** PFS of the 24 R/R PTCL patients. **(F)** DOR of the responders. **(G)** OS of the 24 R/R PTCL patients. H and **(I)** PFS and OS of patients with best response of CR and PR compared with that of SD and PD.

Twenty-four R/R PTCL patients were treated with everolimus-gemcitabine combination (Dec 2017-Mar 2021), with clinical characteristics summarized in **Table 1**. The ORR was 70.8% (17/24):11 patients achieved CR (45.8%) and 6 patients achieved PR (25.0%) (**Figure 1A**). The response according to subtypes were 100% for ALK-ALCL (n=7, CR:57.1%) and SPTCL (n=2, CR:100%), 71.4% (5/7) for PTCL-NOS (CR:57.1%), 50% (1/2) for ALK+ALCL (PR:50%) and 40% (2/5) for AITL (CR:20%).

**Table 1:** Characteristic of the relapsed/refractory PTCL patients (N=24).

Clinical feature	N	%
Age		
median(range)year	48(26-65)	
Gender		
male	16	66.7
female	8	33.3
Stage		
I+II	4	16.7
III+IV	20	83.3
Pathology subtypes		
PTCL-NOS	7	29.2
ALK-ALCL	7	29.2
AITL	5	20.8
SPTCL	2	8.3
ALK+ALCL	2	8.3
MF	1	4.2
B symptoms(presence)	11	45.8
Bulky(presence)	2	8.3
Bone marrow involvement(presence)	4	16.7
Extranodal involvement(presence)	16	66.7
LDH (elevation)	14	58.3
International prognostic index		
1	4	16.7
2-4	20	83.3
Lines of prior treatment (median, range)	2(2-4)	
1	13	54.2
2	6	25
≥3	5	20.8
Prior HSCT	3	12.5
Completed cycles of G+E [median(range)]	4(1-6)	
1	3	12.5
2	3	12.5
3	1	4.2
4	5	20.8
5	1	4.2
6	11	45.8
HSCT after G+E	7	29.2

PTCL-NOS, PTCL-not otherwise specified; AITL, angioimmunoblastic T-cell lymphoma; ALK-ALCL, anaplastic lymphoma kinase (ALK) negative anaplastic large cell lymphoma; ALK+ALCL, anaplastic lymphoma kinase (ALK) positive anaplastic large cell lymphoma; SPTCL, subcutaneous panniculitis-like T-cell lymphoma; MF, mycosis fungoides; LDH, lactic dehydrogenase; G+E, gemcitabine plus everolimus; HSCT, hematopoietic stem cell transplantation.

Median time to response was 1.7 months. With median follow-up of 22.3 months (95% CI 5.7–39.0), median progression-free survival (PFS) was 9.9 months (95% CI 0–20.81), median duration of response (DOR) was 16.8 months, and median overall survival (OS) was 19.2 months (95% CI 4.16–34.24) (**Figures 1B, E–G**). Patients with CR/PR had significantly longer PFS (18.7 vs. 1.2 months, P = 0.0007) and OS (30.9 vs. 5.8 months, P = 0.021) than those with stable/progressive disease (**Figures 1H, I**). Representative cases achieving CR after 2 cycles are shown in **Figures 1C, D**.

Of the 24 patients, 12 completed six treatment cycles, with three proceeding to transplantation. Among the 11 patients who achieved CR, five did not receive transplantation. Three of these five had a prior transplantation history, while the remaining two (aged 65 and 66 years, respectively) declined the procedure. A total of 6 patients with CR underwent hematopoietic stem cell transplantation, comprising 2 cases of ALK-ALCL, 2 cases of PTCL-NOS, 1 case of AITL, and 1 case of SPTCL. One PTCL-NOS patient received allogeneic transplantation but succumbed to pulmonary infection, and this case was included in the 6 transplant recipients (**Table 2**).

**Table 2:** Clinical features of combination therapy provided in all population (N=24).

Patients	Gender	Age	Diagnose	The lines of G+E treatment	N. of cycles of G+E	Consolidative HSCT followed by G+E
Patient#1	female	39	PTCL-NOS	3	6	No
Patient#2	male	48	ALK- ALCL	2	6	ASCT
Patient#3	male	31	ALK- ALCL	4	6	ASCT
Patient#4	male	47	AITL	4	2	No
Patient#5	male	30	PTCL-NOS	2	4	allo-SCT
Patient#6	female	66	AITL	2	6	No
Patient#7	male	35	PTCL-NOS	2	5	ASCT
Patient#8	female	47	AITL	2	3	No
Patient#9	female	48	PTCL-NOS	4	2	No
Patient#10	female	27	SPTCL	2	6	No
Patient#11	male	55	AITL	3	1	No
Patient#12	female	65	ALK-ALCL	3	6	No
Patient#13	male	49	PTCL-NOS	2	1	No
Patient#14	female	60	AITL	4	4	ASCT
Patient#15	male	51	PTCL-NOS	2	6	ASCT
Patient#16	male	37	ALK+ ALCL	3	4	No
Patient#17	male	65	ALK- ALCL	3	6	No
Patient#18	male	48	MF	3	6	No
Patient#19	female	45	SPTCL	4	4	ASCT
Patient#20	male	53	ALK- ALCL	2	6	No
Patient#21	male	60	ALK- ALCL	2	4	No
Patient#22	male	59	PTCL-NOS	2	6	No
Patient#23	female	54	ALK+ ALCL	2	1	No
Patient#24	male	55	ALK-ALCL	2	6	No
HSCT (N=7) vs. no HSCT (N=17)						
PFS (median) 18.7 vs. 5.7 months *HR* 0.755(95%CI: 0.299-1.910) *P*=0.562						
OS (median) 43.0 vs. 12.0 months *HR* 0.519(95%CI: 0.194-1.380) *P*=0.191						

PTCL-NOS, PTCL-not otherwise specified; AITL, angioimmunoblastic T-cell lymphoma; ALCL, anaplastic lymphoma kinase (ALK) negative anaplastic large cell lymphoma; ALK+ALCL, anaplastic lymphoma kinase (ALK) positive anaplastic large cell lymphoma; SPTCL, subcutaneous panniculitis-like T-cell lymphoma; MF, mycosis fungoides; G+E, gemcitabine plus everolimus; HSCT, hematopoietic stem cell transplantation; ASCT, autologous stem cell transplantation; allo-SCT, allogeneic stem cell transplantation; PFS, progression-free survival; OS, overall survival; patient #1, patient #10 and patient #24 received ASCT before G+E treatment.

### Toxicity profile

Grade ≥1 adverse events (AEs) occurred in 20 patients (83.3%), with grade 3–4 AEs in 13 (54.2%). Most common AEs were anemia (83.3%), neutropenia (66.7%), and thrombocytopenia (66.7). Grade 3–4 neutropenia (13 patients, 25% grade 4), thrombocytopenia (11 patients, 37.5% grade 4), and anemia (7 patients, 12.5% grade 4) are detailed in **Supplementary Table S1**.

### AKT-mTOR pathway activation does not predict response

Immunohistochemistry for p-AKT (Ser473) and p-p70S6K (Thr421/Ser424) was performed in 16 patients with available tissues. p-AKT positivity (≥10% tumor cells) was observed in 6 (37.5%), and p-p70S6K in 13 (81.3%) (**Supplementary Figures S1A–F, I**). ORR was 83.3% (p-AKT+) vs. 60.0% (p-AKT−) and 69.2% (p-p70S6K+) vs. 69.2% (p-p70S6K−), with no significant association with PFS/OS (**Supplementary Figures S1G, H**).

### *In vitro* sensitivity of PTCL cell lines to monotherapies

**Figure 2:**
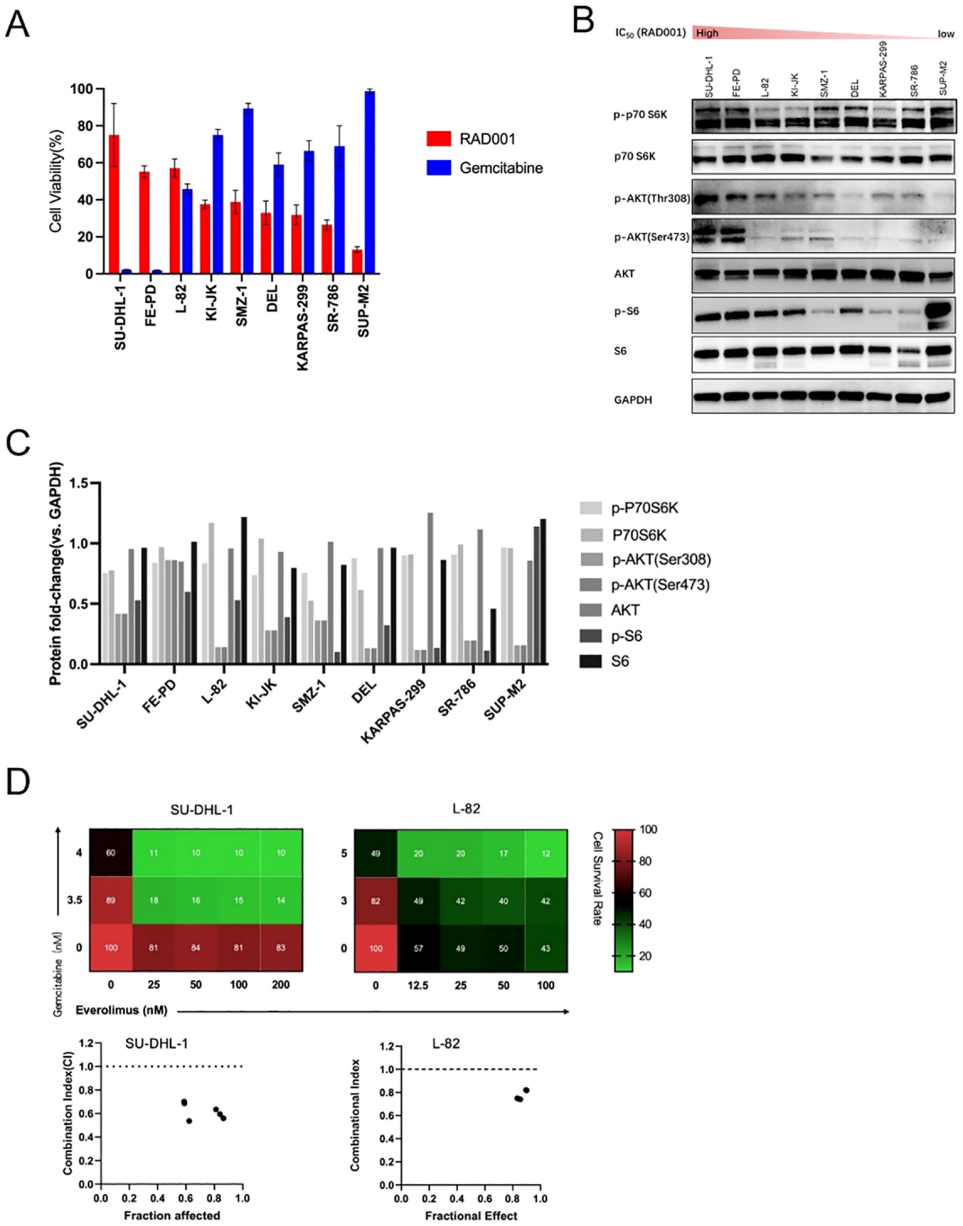
Characterization of PTCL cell lines sensitivity to everolimus and gemcitabine. **(A)** The anti-tumor effect of everolimus (0.25μM) and gemcitabine (0.005μM) alone in nine human PTCL cell lines. **(B)** Examination of the mTOR signaling pathway activation in nine human PTCL cell lines. **(C)** Changes in band intensity of the mTOR signaling pathway in nine human PTCL cell lines. **(D)** (above) Cell survival rate of gemcitabine combined with everolimus (RAD001) under different concentration conditions. (below) Combinational Index (CI) of everolimus and gemcitabine in two cell lines was calculated by Chou-Talalay method (CI values < 1: synergism; CI values > 1: antagonism; CI = 1: additive effect). CI of everolimus and gemcitabine in SU-DHL-1, L-82 cell lines (CI values < 1: synergism).

Everolimus and gemcitabine monotherapy efficacy was evaluated in 9 PTCL cell lines (**Figure 2A**). Everolimus-resistant lines (SU-DHL-1, FE-PD, L-82) showed elevated p-AKT/p-S6 levels (**Figures 2B, C**), with somatic mutations listed in **Supplementary Table S2**.

### Synergistic antitumor activity of the combination

**Figure 3:**
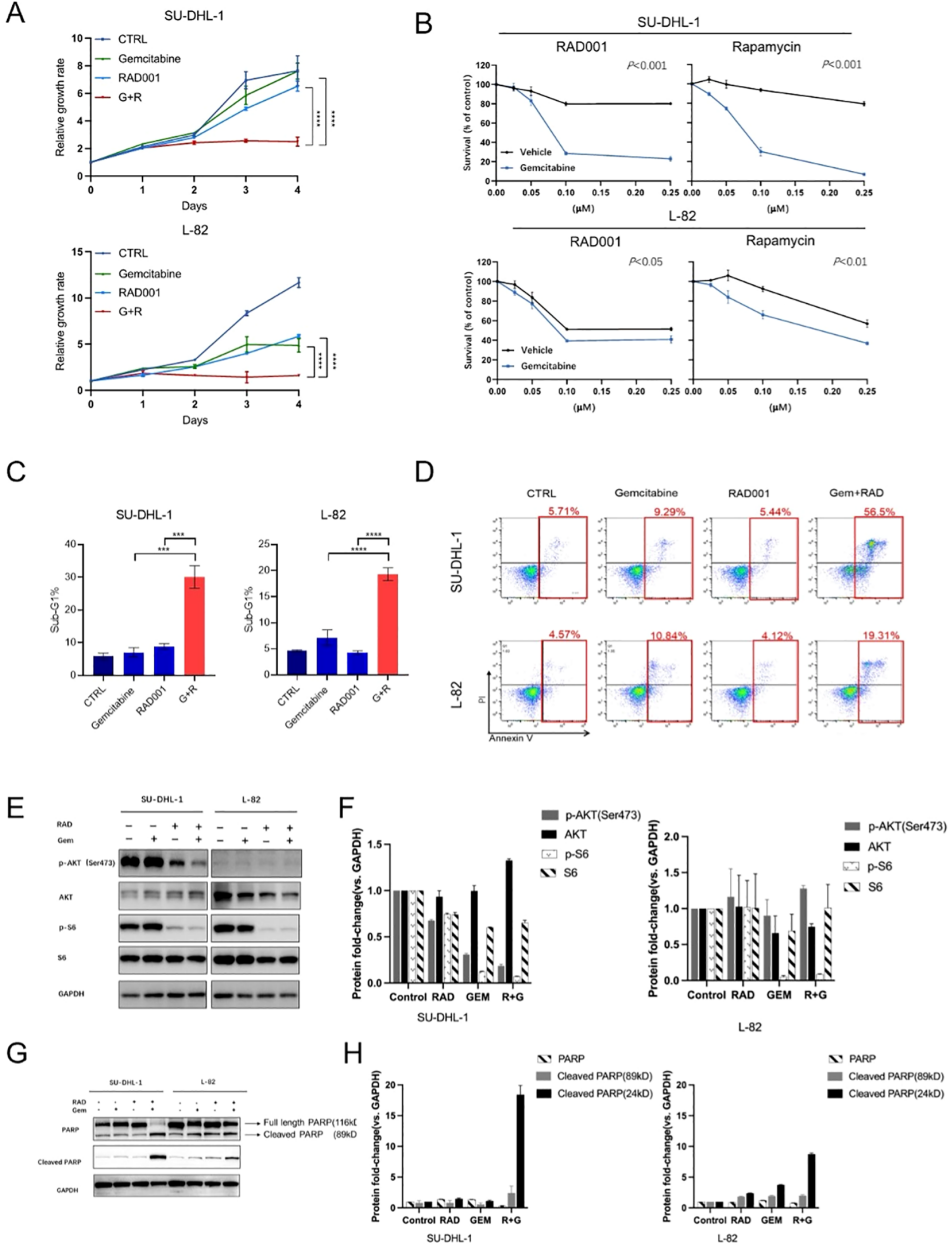
The synergistic effect between everolimus and gemcitabine. **(A)** Growth curve of SUDHL-1 and L-82 cell lines treated with everolimus (0.05μM), gemcitabine (0.0025μM), everolimus (0.05μM) combined with gemcitabine (0.0025μM). **(B)** The synergistic effect between gemcitabine (SU-DHL1: 0.0025μM; L-82: 0.0035μM) and mTOR inhibitors in SUDHL-1 and L-82 cell lines. **(C)** Everolimus (0.05μM) combined with gemcitabine (SU-DHL-1: 0.004μM; L-82: 0.005μM) induced more apoptosis in SUDHL-1 and L-82 cell lines. **(D)** Combination of everolimus and gemcitabine induced significantly more apoptosis as compared to everolimus or gemcitabine alone in SU-DHL-1 and L-82 cell lines. **(E)** The inhibitory effect on the mTOR signaling pathway in L-82 and SU-DHL-1 cells. **(F)** Changes in band intensity of the mTOR signaling pathway in L-82 and SU-DHL-1 cells. **(G)** The combination treatment induced significant PARP cleavage. **(H)** Changes in band intensity of the PARP in L-82 and SU-DHL-1 cells. **(C–F)** use the same dosage for experiments as follows: gemcitabine (SU-DHL-1: 0.004μM; L-82: 0.005μM) and everolimus (0.05μM). All data are presented as mean ± SEM; n=3 independent experiments. *p* values denoted as * (0.01–0.05), (0.001–0.01), (0.0001–0.001), or *** (< 0.0001), *** (0.0001–0.001), or **** (< 0.0001).

Combination index (CI) analysis in everolimus-resistant SU-DHL-1/L-82 showed CI < 1, indicating synergism (**Figure 2D**). The combination synergistically inhibited cell viability/proliferation (**Figures 3A, B**), induced sub-G1 phase accumulation (**Figure 3C**), and promoted apoptosis (**Figure 3D**), validated with rapamycin (another mTOR inhibitor) (**Figure 3B**). Western blotting showed everolimus alone inhibited p-AKT/p-S6, with gemcitabine further enhancing inhibition (**Figures 3E, F**). Combination treatment induced PARP cleavage, confirming increased apoptosis (**Figures 3G, H**).

### MYC pathway suppression drives synergistic efficacy

**Figure 4:**
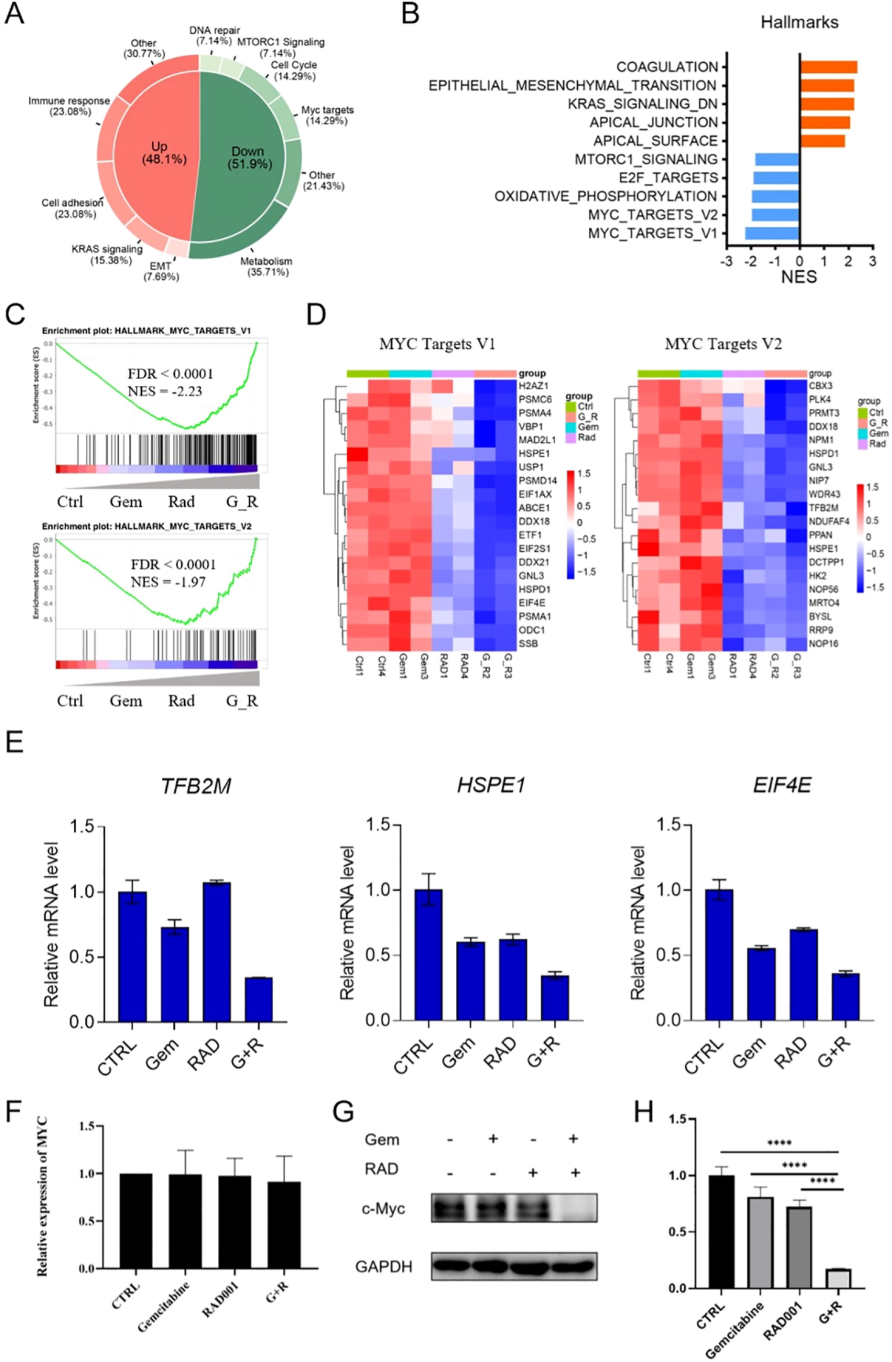
Gemcitabine combined with everolimus suppressed MYC signaling pathway. **(A-C)** The Gene Set Enrichment Analysis (GSEA) of the differential genes in the combination group demonstrated a distinct enrichment of downregulated genes in pathways linked to MYC targets (14.29%). **(D)** MYC targets genes including *TFB2M*, *HSPE1* and *EIF4E* were synergistically suppressed by gemcitabine and everolimus treatment. **(E)** Validation of *TFB2M*, *HSPE1* and *EIF4E* genes in SU-DHL-1 cells by q-PCR. **(F)** There is no significant difference in MYC mRNA level in SU-DHL-1 cells. **(G)** MYC protein was significantly inhibited by the combination treatment. **(H)** Changes in band intensity of MYC in in SU-DHL-1 cells, with GAPDH as control, gemcitabine, everolimus, gemcitabine plus everolimus. All data are presented as mean ± SEM; n=3 independent experiments. * (0.01–0.05), (0.001–0.01), (0.0001–0.001), or *** (< 0.0001), **** (< 0.0001).

RNA-seq in SU-DHL-1 cells revealed MYC target pathways were significantly downregulated by the combination (**Figures 4A–C**). Genes like TFB2M, HSPE1, and EIF4E (MYC targets) were suppressed (**Figure 4D**), confirmed by qPCR (**Figure 4E**). MYC mRNA was unchanged, but protein levels decreased (**Figures 4F–H**), suggesting proteolytic degradation.

**Figure 5:**
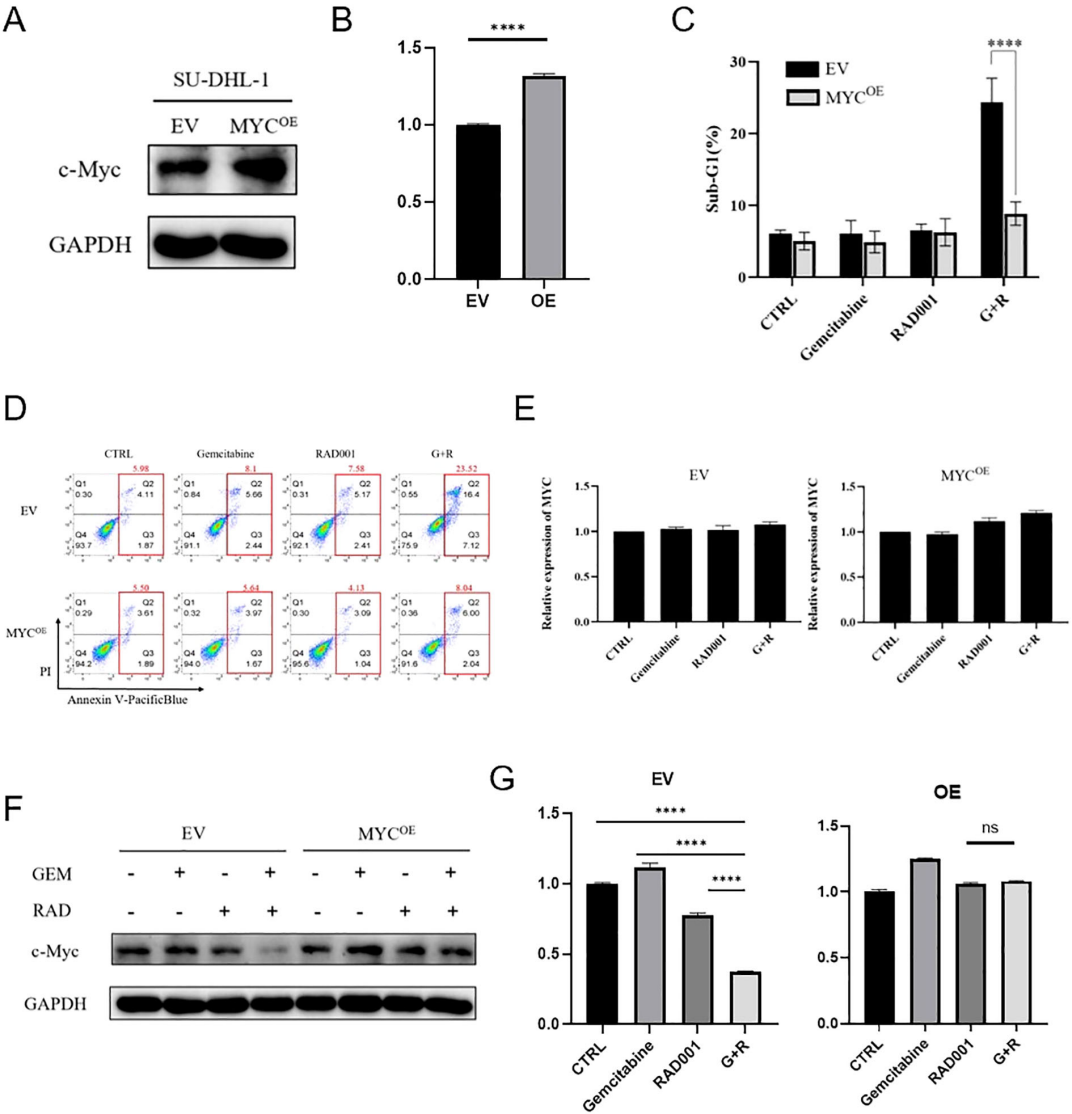
The role of MYC in the synergistic effect of combination. **(A)** MYC was stably transfected in SU-DHL-1 cells. **(B)** Changes in band intensity of MYC in SU-DHL-1-EV (empty-vector) and SU-DHL-1-OE (overexpression) (MYC) cells. **(C)** Analysis of apoptosis unveiled a higher frequency of apoptotic occurrences upon the combined therapeutic regimen as opposed to either everolimus or gemcitabine individually in SU-DHL-1-EV cells when juxtaposed with SU-DHL-1-OE cells. **(D)** Flow cytometry analysis elucidated a notable mitigation of cellular demise in SU-DHL-1-OE cells relative to SU-DHL-1-EV cells upon exposure to the combined therapeutic regimen. **(E)** There were no notable differences detected in MYC mRNA expression levels within the SU-DHL-1-EV or SU-DHL-1-OE cellular cohorts after exposure to the combined therapeutic protocol, as opposed to the effects observed with everolimus or gemcitabine monotherapy. **(F)** Following exposure to the combined therapeutic regimen, the expression of the MYC protein exhibited a decrease in SU-DHL-1-EV cells, whereas the levels of MYC protein remained unaltered in SU-DHL-1-OE cells. **(G)** Changes in the band intensity of the MYC protein were assessed in both SU-DHL-1-EV and SU-DHL-1-OE cellular populations after exposure to GAPDH, gemcitabine, everolimus, and the combined treatment of gemcitabine and everolimus. (gemcitabine: 0.004μM, everolimus: 0.05μM). All data are presented as mean ± SEM; n=3 independent experiments. * (0.01–0.05), (0.001–0.01), (0.0001–0.001), *** (< 0.0001), **** (< 0.0001).

MYC-overexpressing SU-DHL-1 cells (SU-DHL-1-OE) showed rescued viability upon combination treatment vs. empty-vector controls (SU-DHL-1-EV) (**Figures 5A–D**). MYC mRNA was unaltered (**Figure 5E**), while MYC protein was degraded in SU-DHL-1-EV but stable in SU-DHL-1-OE (**Figures 5F, G**). These data indicate the combination abrogates MYC signaling to drive synergistic cytotoxicity.

## Discussion

Given the poor prognosis associated with relapsed or refractory (R/R) peripheral T-cell lymphoma (PTCL) and the significant challenges arising from its rarity and pronounced heterogeneity, there is a critical and unmet need for the development of novel therapeutic strategies that are both effective and exhibit reduced toxicity. While monotherapies employing the mTOR inhibitor everolimus or the antimetabolite gemcitabine have shown limited clinical benefit in R/R PTCL, our preclinical investigations demonstrate that their combined administration produces synergistic effects by inhibiting cell proliferation and enhancing apoptosis in everolimus-resistant PTCL cell lines. This synergistic interaction appears to be mediated, at least partially, through the downregulation of the MYC signaling pathway, thereby underscoring the potential clinical relevance of this combination therapy in improving treatment outcomes for patients with R/R PTCL.

Preclinical studies have consistently demonstrated the *in vitro* antineoplastic efficacy of everolimus across a range of hematological malignancies. Clinically, treatment with everolimus as a single agent has yielded overall response rates (ORRs) between 20% and 47% in patients with relapsed or refractory Hodgkin lymphoma (HL) and non-Hodgkin lymphoma (NHL) (18–20). Nevertheless, data specifically addressing relapsed or refractory peripheral T-cell lymphoma (R/R PTCL) are limited. A phase II clinical trial investigating the administration of oral everolimus at doses ranging from 5 to 10 mg daily in a cohort of 13 patients with relapsed or refractory NHL—comprising 9 individuals with B-cell lymphoma and 4 with PTCL—demonstrated an ORR of 31% among the B-cell lymphoma subgroup. In contrast, no objective responses were observed within the PTCL subgroup. This disparity may be attributable to suboptimal dosing strategies or inherent resistance mechanisms characteristic of T-cell malignancies (19). In an expanded cohort of patients with relapsed or refractory peripheral T-cell lymphoma (R/R PTCL), daily monotherapy with 10 mg of everolimus resulted in a modest ORR of 44%, accompanied by a complete response (CR) rate of only 6.3%, and a median progression-free survival (PFS) of 4.1 months (11). Taken together, these findings underscore the imperative for combinatorial therapeutic approaches aimed at improving CR rates, extending the duration of sustained responses, and inhibiting disease progression within this patient cohort (21).

Significantly, this investigation represents the first clinical assessment of the efficacy and safety of the everolimus-gemcitabine combination therapy in patients with relapsed or refractory peripheral T-cell lymphoma (R/R PTCL). The treatment regimen yielded promising clinical results, achieving an ORR of 70.8% and a median PFS of 9.9 months. Notably, among the 11 patients who attained CR, seven proceeded to hematopoietic stem cell transplantation (HSCT), comprising six autologous HSCT (ASCT) and one allogeneic HSCT (allo-SCT). This transition represents a critical therapeutic milestone, as HSCT remains the sole curative intervention for selected patients with R/R PTCL. Furthermore, three patients deemed ineligible for HSCT exhibited sustained disease control, with PFS durations ranging from 17.2 to 22.2 months. These findings surpass previously reported outcomes associated with everolimus or gemcitabine monotherapies, as well as other established combination regimens for R/R PTCL (**Table 3**), thereby reinforcing the potential of this combination as a valuable therapeutic strategy.

**Table 3:** Clinical efficacy of agents in relapsed or refractory PTCL.

regimens	N	ORR (%)	CRR (%)	mPFS (months)	mOS (moths)	Ref.
Single agent						
Romidepsin	N=130	25	15	4.0	11.3	(4)
Belinostat	N=129	25.8	10.8	1.6	7.9	(5)
Pralatrexate	N=111	29	11	3.5	14.5	(6)
BV	N=34	41	24	2.6		(7)
Duvelisib	N=16	50	18.8	8.3	8.4	(10)
Everolimus	N=16	44	6.3	4.1	10.2	(11)
Gemcitabine	N=39	50	23	NR		(12)
Chidamide	N=256	39	10.5	129days		(22)
Bendamustine	N=60	50	28	3.6	6.2	(23)
Lenalidomide	N=39	26	8	4.0	12.0	(24)
Combination therapy regimens						
romidepsin+ 5-azacytidine	N=13	54	38	8.0	20.6	(25)
romidepsin+pralatrexate	N=14	71	40	4.4	12.4	(26)
romidepsin+lenalidomide	N=19	58	11	13.5weeks	NR	(27)
panobinostat+bortezomib	N=23	43	22	2.59	9.9	(28)
romidepsin+pembrolizumab	N=14	50	35.7	NR		(29)
romidepsin+gemcitabine	N=20	30	15	2-year PFS11.2%	2-year OS 50%	(30)
copanlisib+gemcitabine	N=25	72	32	6.9	NR	(31)
bortezomib+gemcitabine	N=16	36	27	NR	NR	(32)
everolimus+gemcitabine	N=24	70.8	45.8	9.9	19.2	*

N, number; ORR, objective response rate; CRR, complete response rate; PFS, progression free survival. OS, overall survival; m, median; BV, brentuximab vedotin; NR, not reported; *, our study

The variability in treatment responses observed among distinct PTCL subtypes may be attributed to differences in the baseline activation levels of the mTOR signaling pathway, which constitutes a principal therapeutic target of everolimus. Previous research has indicated that biomarkers predictive of sensitivity to everolimus are strongly associated with the activity of the mTOR pathway (33). For example, anaplastic large cell lymphoma (ALCL) generally demonstrates increased expression of phosphorylated AKT (p-AKT) relative to peripheral T-cell lymphoma not otherwise specified (PTCL-NOS) and angioimmunoblastic T-cell lymphoma (AITL). Moreover, elevated levels of p-AKT have been associated with poorer overall survival (OS) and PFS in patients with PTCL (34). While phosphorylation of the ribosomal protein S6 (a downstream effector of mTOR) has been proposed as a putative biomarker for predicting response to mTOR inhibitors (35), our analysis failed to identify a significant correlation between pretreatment p-AKT or phosphorylated p70S6K (p-p70S6K) levels and treatment efficacy (ORR, PFS, or OS). This lack of correlation could be due to several reasons: first, the activation of the mTOR pathway is dynamic and influenced by the environment, so a single tissue sample taken before treatment might not accurately represent its true status; second, other signaling pathways like JAK-STAT and NF-κB may affect treatment response independently of mTOR; third, the limited size of our study group may reduce the statistical power needed to identify subtle relationships. Therefore, it is worthwhile to investigate other potential predictive biomarkers, such as the expression levels of MYC and its downstream target genes, since our preclinical research has demonstrated that inhibiting MYC is linked to enhanced treatment effects.

Regarding safety, although patients who had undergone extensive prior treatments (such as multiple chemotherapy regimens or hematopoietic stem cell transplantation) demonstrated a higher frequency of hematological adverse events, including grade 3–4 neutropenia and thrombocytopenia, the overall safety profile of the everolimus and gemcitabine combination was considered manageable with the implementation of appropriate supportive care measures. However, this study is subject to several limitations inherent to its retrospective nature. Firstly, the relatively small sample size (n=24) may introduce selection bias, as all participants were recruited from a single institution, potentially limiting the generalizability of the findings to the wider relapsed/refractory peripheral T-cell lymphoma (R/R PTCL) population. Secondly, the absence of a control group precludes direct comparison with established standard-of-care treatments, thereby hindering definitive conclusions regarding the superiority of the investigated combination. Thirdly, the availability of long-term follow-up data is restricted, particularly concerning late-onset toxicities and the durability of remission in patients who did not receive hematopoietic stem cell transplantation.

Consistent with previous study (11), everlimus monotherapy exhibited variable *in vitro* activity, with resistance observed in certain cell lines. To investigate potential synergistic interactions between everolimus and gemcitabine, we performed detailed dose-response studies using relapsed/refractory peripheral T-cell lymphoma (R/R PTCL) cell line models. These analyses assessed cell viability, proliferation, and apoptosis following treatment with each agent individually and in combination. Employing the Chou-Talalay method to determine combination indices (CIs), we identified synergistic effects (CI < 1) in four PTCL cell lines, with notably pronounced synergy in two models exhibiting resistance to everolimus. Mechanistically, gemcitabine—a cell cycle-specific agent that impedes DNA replication through inhibition of ribonucleotide reductase—enhanced the anti-proliferative activity of everolimus, an mTOR inhibitor, by augmenting cell cycle arrest and inducing apoptosis. Furthermore, RNA sequencing analyses demonstrated that the combination treatment significantly downregulated genes associated with MYC signaling pathways. Complementary Western blot analyses confirmed that this combination promoted degradation of the MYC protein without affecting its transcriptional expression levels. Given the well-established role of aberrant MYC expression in PTCL pathogenesis, disease progression, and poor prognosis (36–38), targeting MYC protein stability through this combinatorial approach may represent a novel and effective therapeutic strategy. Future studies are needed to delineate the precise molecular mechanisms underlying MYC downregulation by the everolimus-gemcitabine combination, such as the potential involvement of E3 ubiquitin ligases or proteasomal degradation pathways.

## Conclusion

The everolimus-gemcitabine combination shows promising efficacy and manageable toxicity in relapsed/refractory PTCL, with an ORR of 70.8% and median PFS of 9.9 months. Mechanistically, this combination synergistically inhibits PTCL cell growth by abrogating the MYC signaling pathway, as validated by transcriptomic and functional studies. Further research is warranted to explore translational potential and optimize MYC-targeted strategies in clinical settings.

## Data availability statement

The datasets presented in this study can be found in online repositories. The names of the repository/repositories and accession number(s) can be found in the article/**Supplementary Material**.

## Ethics statement

This study was performed according to the Declaration of Helsinki principles and approved by the Institutional Review Board of Guangdong Provincial People’s Hospital (No. KY2020-068-01, KY-Z-2020-670- 02). All patients provided written informed consent.

## Supplementary material

Supplementary Figure 1 Immunohistochemistry analysis of p-AKT and p-p70S6K in R/R PTCL patients. **(A–C)** Immunohistochemistry of p-AKT protein expression:1+, ++ and +++. **(D–F)**. Immunohistochemistry of p70S6K protein expression: 1+, ++ and +++. **(G, H)**. PFS of patients according to p-AKT and p70S6K expression. (I). Expression of p-AKT and p70S6K in different subtypes.

Supplementary Figure 2 Characterization of SMZ-1 and SUP-M2 cell lines sensitivity to everolimus and gemcitabine. Combinational Index (CI) of everolimus and gemcitabine in SMZ-1 and SUP-M2 cell lines were calculated by Chou-Talalay method (CI value<1: synergism; CI values>1: antagonism; CI values=1: additive effect). Combinational Index of everolimus and gemcitabine in SMZ-1(A) and SUP-M2 (B) cell lines (CI values>1: antagonism).
