# Supplementary figures and images for: Synergistic anti-tumor activity of the mTOR inhibitor everolimus and gemcitabine for relapsed/refractory peripheral T cell lymphoma
Source: Front Immunol. 2025 Nov 28;16:1683550. doi: 10.3389/fimmu.2025.1683550 (PMC12698551; doi:10.3389/fimmu.2025.1683550)

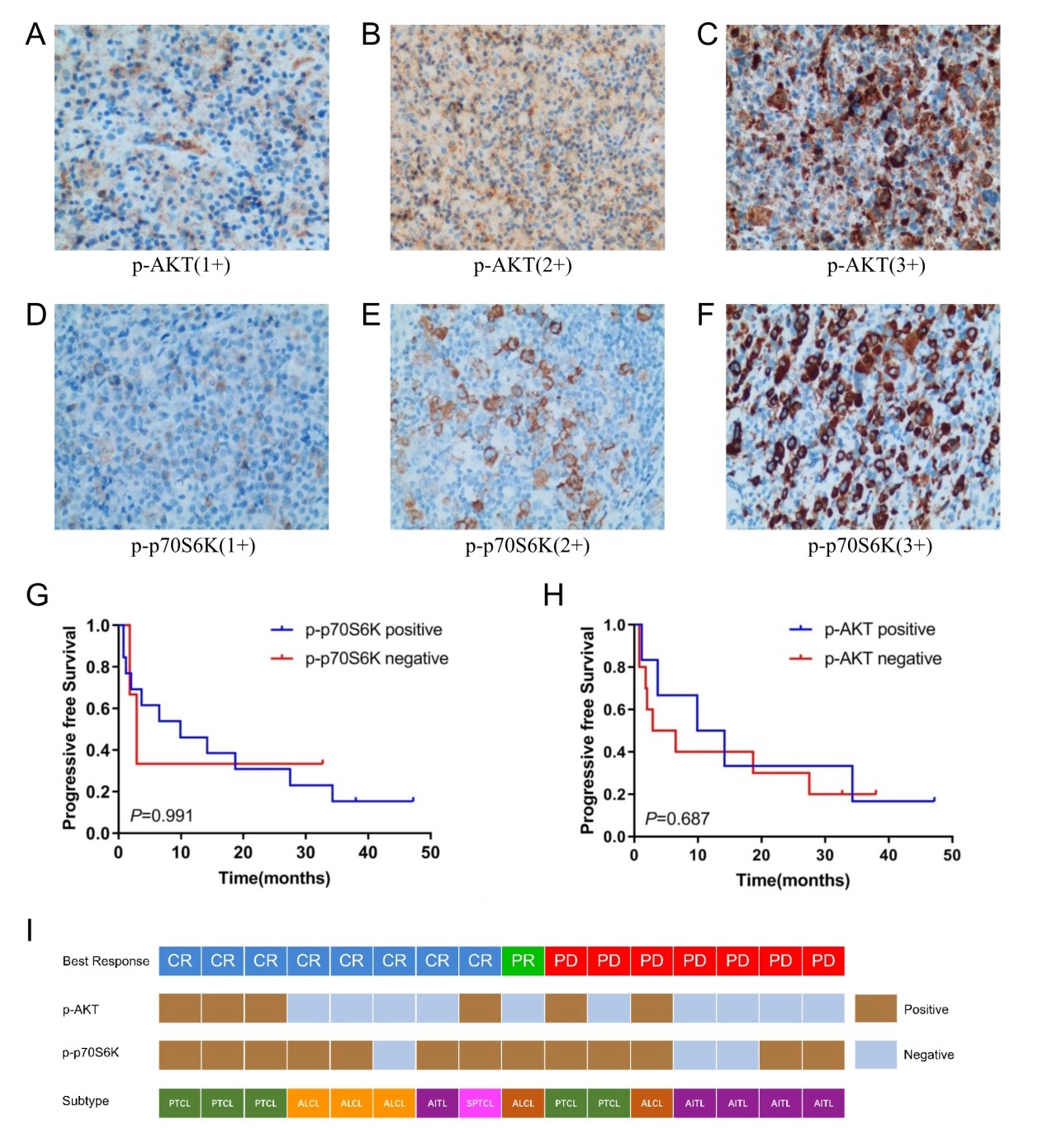

Supplement: Supplementary Figure 1 — Immunohistochemistry analysis of p-AKT and p-p70S6K in R/R PTCL patients. (A–C) Immunohistochemistry of p-AKT protein expression:1+, ++ and +++. (D–F). Immunohistochemistry of p70S6K protein expression: 1+, ++ and +++. (G, H). PFS of patients according to p-AKT and p70S6K expression. (I). Expression of p-AKT and p70S6K in different subtypes. [file Image1.tiff]

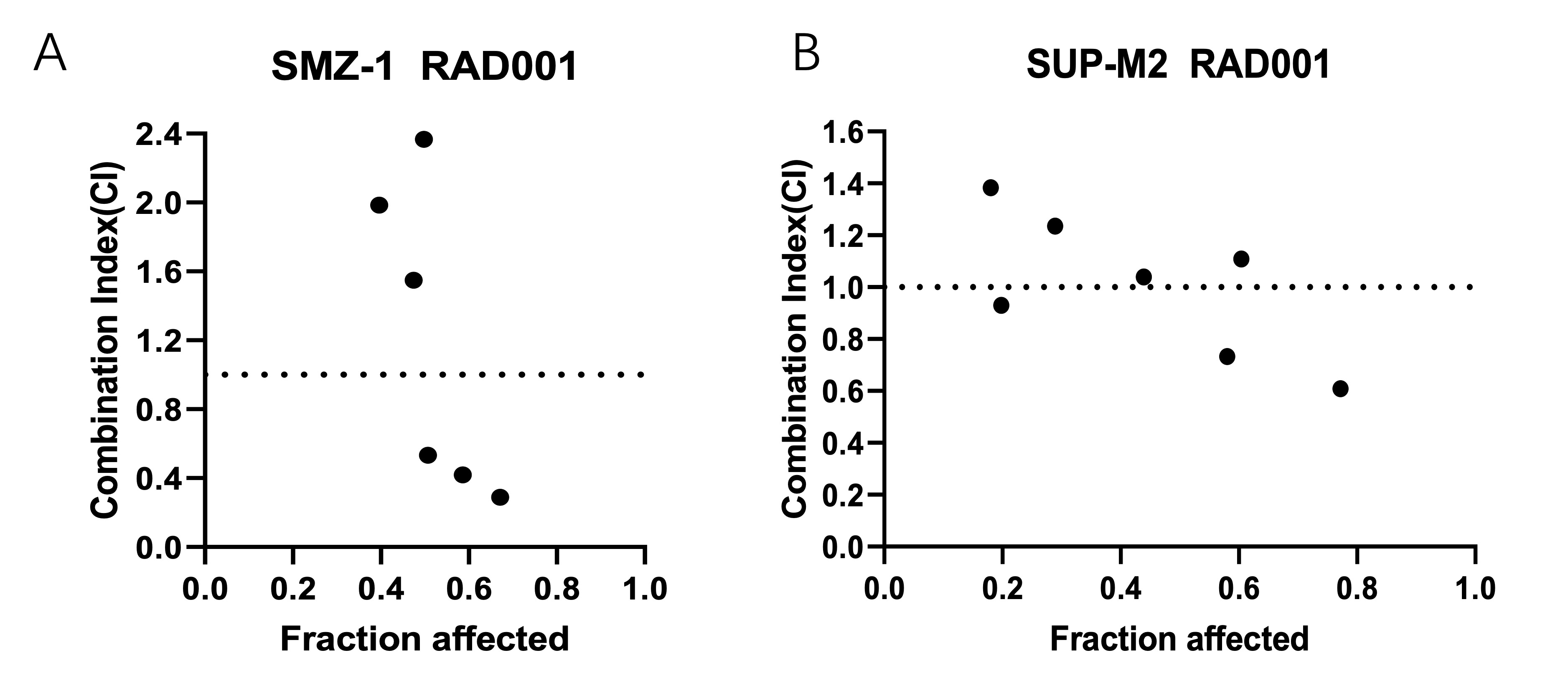

Supplement: Supplementary Figure 2 — Characterization of SMZ-1 and SUP-M2 cell lines sensitivity to everolimus and gemcitabine. Combinational Index (CI) of everolimus and gemcitabine in SMZ-1 and SUP-M2 cell lines were calculated by Chou-Talalay method (CI value<1: synergism; CI values>1: antagonism; CI values=1: additive effect). Combinational Index of everolimus and gemcitabine in SMZ-1(A) and SUP-M2 (B) cell lines (CI values>1: antagonism). [file Image2.tiff]
