# Supplementary material for: Synergistic anti-tumor activity of the mTOR inhibitor everolimus and gemcitabine for relapsed/refractory peripheral T cell lymphoma
Source: Front Immunol. 2025 Nov 28;16:1683550. doi: 10.3389/fimmu.2025.1683550 (PMC12698551; doi:10.3389/fimmu.2025.1683550)
Supplement: Supplementary file 3 [file Table1.docx]

**Table S1 Adverse events in patients undergoing gemcitabine and everolimus combination treatment**

| Adverse events | All grades  (N, %) | Grade 1-2  (N, %) | Grade3  (N, %) | Grade4  (N, %) |
| --- | --- | --- | --- | --- |
| Neutropenia  Anemia  Thrombocytopenia  Fever  Pneumonia  Serum ALT elevation  Serum AST elevation  Fatigue  Stomatitis  Anorexia  Diarrhea  Nausea  Skin infection  Rash  Pleural effusion  Interstitial pneumonia | 16(66.7)  20(83.3)  16(66.7)  8(33.3)  8(33.3)  7(29.2)  6(25.0)  6(25.0)  4(16.7)  4(16.7)  3(12.5)  3(12.5)  3(12.5)  1(4.2)  1(4.2)  0(0.0) | 3(12.5)  13(54.2)  5(20.8)  5(20.8)  3(12.5)  6(25.0)  5(20.8)  4(16.7)  1(4.2)  3(12.5)  2(8.3)  3(12.5)  3(12.5)  0(0.0)  1(4.2)  0(0.0) | 7(29.2)  4(16.6)  2(8.3)  3(12.5)  4(16.6)  1(4.2)  1(4.2)  2(8.3)  3(12.5)  1(4.2)  1(4.2)  0(0.0)  0(0.0)  1(4.2)  0(0.0)  0(0.0) | 6(25.0)  3(12.5)  9(37.5)  0(0.0)  1(4.2)  0(0.0)  0(0.0)  0(0.0)  0(0.0)  0(0.0)  0(0.0)  0(0.0)  0(0.0)  0(0.0)  0(0.0)  0(0.0) |

ALT: Alanine transaminase; AST: Aspartate aminotransferase
