# Supplementary material for: Synergistic anti-tumor activity of the mTOR inhibitor everolimus and gemcitabine for relapsed/refractory peripheral T cell lymphoma
Source: Front Immunol. 2025 Nov 28;16:1683550. doi: 10.3389/fimmu.2025.1683550 (PMC12698551; doi:10.3389/fimmu.2025.1683550)
Supplement: Supplementary file 4 [file Table2.docx]

**Table S2 Somatic mutation in nine PTCL cell lines (COSMIC)**

| Cell lines | Mutation (Type) | PIK3CA | | PTEN | AKT |
| --- | --- | --- | --- | --- | --- |
| SU-DHL-1 | TP53(Missense) | | N/A | N/A | N/A |
| FE-PD | JAK1(Missense) /STAT3(Missense) | | N/A | N/A | N/A |
| L-82 | N/A | | N/A | N/A | N/A |
| KI-JK | N/A | | N/A | N/A | N/A |
| SMZ-1 | N/A | | N/A | N/A | N/A |
| DEL | N/A | | N/A | N/A | N/A |
| KARPAS-299 | TP53(Missense) /ALK(Missense) | | N/A | No | N/A |
| SR-786 | TP53(Missense) | | N/A | No | N/A |
| SUP-M2 | ALK(Missense) | | N/A | N/A | N/A |

N/A: no available data
